# Supplementary material for: Molecular Detection of Trypanosoma spp. in Questing and Feeding Ticks (Ixodidae) Collected from an Endemic Region of South-West Australia
Source: Pathogens. 2021 Aug 16;10(8):1037. doi: 10.3390/pathogens10081037 (PMC8398035; doi:10.3390/pathogens10081037)
Supplement: Supplementary file 1 [file pathogens-10-01037-s001.zip › pathogens-1296594-supplementary.pdf]

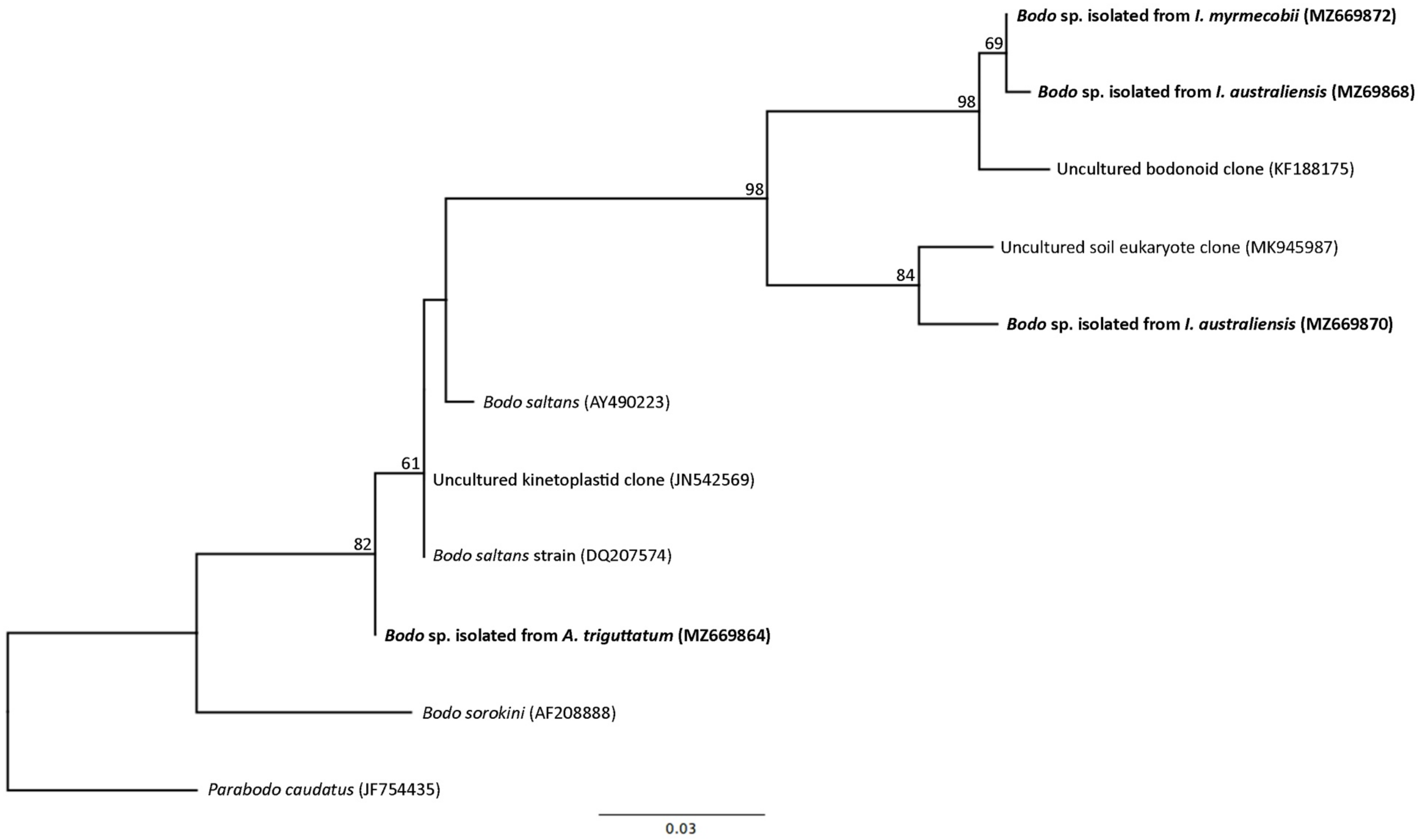

Supplementary Figure S1. Representative ML phylogenetic tree constructed from *Bodo* 18S rRNA partial sequences **(243 bp).** Numbers represent bootstrap support generated from 1000 replications. Values above 60% are indicated. GenBank accession numbers in brackets. Sequences from this study are in bold type.

Supplementary Table S1. *Trypanosoma* and *Bodo* sequences generated in [22] and the present study.

Sample ID	*Trypanosoma* sp.	Tick species	Tick life stage	Status	Accession number
2018QT9	*T. copemani*	*I. myrmecobii*	Female	Questing	MW881302
2018QT12	*T. copemani*	*A. triguttatum*	Nymph	Questing	MW881303
2018QT33	*T. copemani*	*A. triguttatum*	Nymph	Questing	MW881304
2018QT34	*T. copemani*	*A. triguttatum*	Nymph	Questing	MW881305
2018QT35	*T. copemani*	*A. triguttatum*	Nymph	Questing	MW881306
2018QT45	*T. copemani*	*A. triguttatum*	Female	Questing	MW881307
2018QT57	*T. copemani*	*A. triguttatum*	Nymph	Questing	MW881308
2018QT65	*T. copemani*	*A. triguttatum*	Nymph	Questing	MW881309
2018QT79	*T. copemani*	*A. triguttatum*	Nymph	Questing	MW881310
2018QT75	*T. copemani*	*A. triguttatum*	Male	Questing	MW881311
2018QT78	*T. copemani*	*A. triguttatum*	Female	Questing	MW881312
2018QT78	*T. copemani*	*A. triguttatum*	Female	Questing	MW881313
2018QT86	*T. copemani*	*A. triguttatum*	Male	Questing	MW881314

2018QT109	*T. copemani*	*A. triguttatum*	Nymph	Questing	MW881315
2018FT26	*T. copemani*	*I. australiensis*	Female	Feeding	MW881316
2018FT31b	*T. copemani*	*I. australiensis*	Nymph	Feeding	MW881317
2018FTJ5	*T. copemani*	*I. tasmani*	Nymph	Feeding	MW881318
2018FTJ9	*T. copemani*	*I. myrmecobii*	Nymph	Feeding	MW881319
2018FTJ11	*T. copemani*	*I. woyliei*	Female	Feeding	MW881320
2018FT31	*T. copemani*	*I. tasmani*	Nymph	Feeding	MW881321
2018QT26	*T. noyesi*	*A. triguttatum*	Nymph	Questing	MW079253
2018QT34	*T. noyesi*	*A. triguttatum*	Nymph	Questing	MW079254
2018QT50	*T. noyesi*	*A. triguttatum*	Nymph	Questing	MW079255
2018QT51	*T. noyesi*	*A. triguttatum*	Nymph	Questing	MW079256
2018QT66	*T. noyesi*	*A. triguttatum*	Nymph	Questing	MW079257
2018QT69	*T. noyesi*	*A. triguttatum*	Nymph	Questing	MW079258
2018FT4	*T. noyesi*	*A. triguttatum*	Nymph	Feeding	MW881322
2018QT13	*T. vegrandis/T. gilletti*	*I. australiensis*	Male	Questing	MW881323

2018QT32	*T. vegrandis/T. gilletti*	*A. triguttatum*	Nymph	Questing	MW881324
2018QT62	*T. vegrandis/T. gilletti*	*A. triguttatum*	Male	Questing	MW881325
2018QT63	*T. vegrandis/T. gilletti*	*A. triguttatum*	Female	Questing	MW881326
2018QT77	*T. vegrandis/T. gilletti*	*A. triguttatum*	Nymph	Questing	MW881327
2018QT84	*T. vegrandis/T. gilletti*	*A. triguttatum*	Nymph	Questing	MW881328
2018QT91	*T. vegrandis/T. gilletti*	*A. triguttatum*	Nymph	Questing	MW881329
2018QT100	*T. vegrandis/T. gilletti*	*A. triguttatum*	Nymph	Questing	MW881330
2018QT104	*T. vegrandis/T. gilletti*	*A. triguttatum*	Nymph	Questing	MW881331
2018QT111	*T. vegrandis/T. gilletti*	*I. australiensis*	Female	Questing	MW881332
2018QT118	*T. vegrandis/T. gilletti*	*A. triguttatum*	Nymph	Questing	MW881333
2018QT122	*T. vegrandis/T. gilletti*	*I. australiensis*	Nymph	Questing	MW881334
2018FT6a	*T. vegrandis/T. gilletti*	*Amblyomma* spp.	Larva	Feeding	MW881335
2018FT7b	*T. vegrandis/T. gilletti*	*Amblyomma* spp.	Larva	Feeding	MW881336
2018FT30a	*T. vegrandis/T. gilletti*	*I. tasmani*	Nymph	Feeding	MW881337
2018FT31a	*T. vegrandis/T. gilletti*	*I. australiensis*	Nymph	Feeding	MW881338

2018FT55	*T. vegrandis*/*T. gilletti*	*I. myrmecobii*	Female	Feeding	MW881339
2018FTJ7	*T. vegrandis*/*T. gilletti*	*I. tasmani*	Nymph	Feeding	MW881340
2018FTJ14	*T. vegrandis*/*T. gilletti*	*I. woyliei*	Female	Feeding	MW881341
2018FTJ19	*T. vegrandis*/*T. gilletti*	*I. myrmecobii*	Female	Feeding	MW881342
2018FTJ21	*T. vegrandis*/*T. gilletti*	*I. tasmani*	Nymph	Feeding	MW881343
2018FTJ26	*T. vegrandis*/*T. gilletti*	*I. australiensis*	Nymph	Feeding	MW881344
2018FTJ28	*T. vegrandis*/*T. gilletti*	*Amblyomma* spp.	Larva	Feeding	MW881345
2018QT20	*Trypanosoma* sp. ANU2	*A. triguttatum*	Nymph	Questing	MW881346
2018FTJ20	*Trypanosoma* sp. ANU2	*I. myrmecobii*	Female	Feeding	MW881347
2018FTJ25	*Trypanosoma* sp. ANU2	*I. myrmecobii*	Female	Feeding	MW881348
2018FTJ29	*Trypanosoma* sp. ANU2	*Ixodes* spp.	Larva	Feeding	MW881349
2018QT3	*Bodo* sp.	*A. triguttatum*	Nymph	Questing	MZ669864
2018QT25	*Bodo* sp.	*A. triguttatum*	Nymph	Questing	MZ669865
2018QT28	*Bodo* sp.	*A. triguttatum*	Nymph	Questing	MZ669866
2018QT37	*Bodo* sp.	*I. myrmecobii*	Female	Questing	MZ669867

2018QT39	*Bodo* sp.	*I. australiensis*	Male	Questing	MZ669868
2018QT44	*Bodo* sp.	*A. triguttatum*	Nymph	Questing	MZ669869
2018QT64	*Bodo* sp.	*I. australiensis*	Nymph	Questing	MZ669870
2018QT72	*Bodo* sp.	*A. triguttatum*	Male	Questing	MZ669871
2018QT90	*Bodo* sp.	*I. myrmecobii*	Female	Questing	MZ669872
2018QT93	*Bodo* sp.	*I. australiensis*	Male	Questing	MZ669873
2018QT96	*Bodo* sp.	*A. triguttatum*	Nymph	Questing	MZ669874
2018QT97	*Bodo* sp.	*A. triguttatum*	female	Questing	MZ669875
2018QT101	*Bodo* sp.	*A. triguttatum*	Nymph	Questing	MZ669876
2018QT106	*Bodo* sp.	*A. triguttatum*	Nymph	Questing	MZ669877
2018QT107	*Bodo* sp.	*I. australiensis*	Male	Questing	MZ669878
2018QT113	*Bodo* sp.	*A. triguttatum*	Nymph	Questing	MZ669879
2018QTW1	*Bodo* sp.	*A. triguttatum*	Nymph	Questing	MZ669880
2018QTJ12	*Bodo* sp.	*A. triguttatum*	Nymph	Questing	MZ669881
2018QTJ17	*Bodo* sp.	*A. triguttatum*	Nymph	Questing	MZ669882
